# GM-CSF and IL-2 as adjuvant enhance the immune effect of protein vaccine against foot-and-mouth disease

**DOI:** 10.1186/1743-422X-8-7

**Published:** 2011-01-09

**Authors:** Can Zhang, Bin Wang, Ming Wang

**Affiliations:** 1 College of Veterinary Medicine, China Agricultural University, Beijing 100193, China; 2 State Key Lab of Agro-Biotechnology and College of Biological Sciences, China Agricultural University, Beijing 100193, China; 3 College of Veterinary Science, Qingdao Agricultural University, Qingdao 266109, China

## Abstract

**Background:**

Cytokines as molecular adjuvant play a critical role in differentiation of effector T cell subsets and in determination of the magnitude of the response after vaccination. In this study, we investigated the effects of GM-CSF and IL-2 as adjuvant on the immune responses of VP1 recombinant protein as a model antigen for foot and mouth disease.

**Results:**

Six expression plasmids were constructed and expressed in *E. coli *BL21. In guinea pigs, the immunological and molecular effects of the fusion proteins were determined by ELISA, LPA, DTH and semi-quantitative Reverse Transcriptase PCR (RT-PCR). The data revealed that IL-2 and GM-CSF as adjuvant of VP1 could stimulate both humoral and cell-mediated immune response. Interestingly, IL-2 and GM-CSF, either as a co-expressed protein or as a mixture of two single proteins, showed much better adjuvant effects than that of single one.

**Conclusions:**

IL-2 and GM-CSF could be used as a potential adjuvant for VP1 and had synergistic effect when co-expressed or mixed with VP1.

## Background

In recent years, there has been significant progress in the development of candidate vaccines against foot and mouth disease virus (FMDV), in the forms of both whole virus and recombinant proteins. Practical application of these vaccines, however, has often been limited by the lack of suitable adjuvant capable of stimulating an appropriate immune response in the absence of adverse reactions.

Many compounds with adjuvant activity have been identified, but none has been emerged as being universally superior [1,2]. Although adjuvant such as alum adjuvant has been widely used with vaccines for many years [3], alum does not effectively augment immune response necessary for a number of new subunit protein or peptide based vaccines [4]. There is a strong need for alternative adjuvants that must not only enhance the immune response but also drive it to achieve the appropriate type of protective immunity in each situation. It is now evident that molecular adjuvant, especially cytokines [5-7], could enhance and modulate the immune responses induced by subunit vaccine. In many studies cytokines were used to reinforce the ability of the subunit vaccine to induce antigen-specific cellular immune response against FMDV [8-11].

IL-2 is one of the most widely used adjuvants for vaccination to stimulate the proliferation and activation of various immune effector cells such as T cells, NK cells, B cells, and macrophages[12,13]. Granulocyte monocyte colony stimulating factor (GM-CSF) is known to stimulate macrophage differentiation and proliferation, and to activate antigen presenting cells [14]. IL-2 and GM-CSF has been used as an effective adjuvant for DNA or peptide based vaccines [15-17].

In this immunization study, we selected IL-2 and GM-CSF as adjuvant for the VP1 subunit vaccine, with an ultimate goal to verify whether these cytokines have the ability to stimulate humoral immune response and cellular immunity for FMDV.

## Results

### Construction of expression plasmids of BoIL-2, BoGM-CSF and VP1

Bovine IL-2 (BoIL-2), Bovine GM-CSF (BoGM-CSF) and VP1 gene were amplified and cloned into pGEX-6P-1 vector by using the restriction enzymes as described before. Each construct was characterized by restriction mapping with one vector band and specific target bands at 405 bp, 450 bp, 378 bp and 669 bp, respectively, followed by DNA sequencing. The results showed that the plasmids of BoIL-2, BoGM-CSF and VP1 were correctly constructed with sequence integrity and right orientation.

### Construction of co-expression plasmids of BoIL-2, BoGM-CSF and VP1

BoIL-2, BoGM-CSF and VP1 gene fragments were amplified and cloned into pGEX-6P-1 vector by using the restriction enzymes as described before. To construct fused products of BoIL-2/BoGM-CSF/VP1, BoIL-2/VP1, BoGM-CSF/VP1, These constructs were characterized by double digestion with the corresponding restriction enzymes and yielded fragments including one vector band and specific target bands, of which 669 bp was expected for the VP1, 405 bp for the BoIL-2, 378 bp for the BoGM-CSF, 1089 bp for the BoIL-2/VP1, 1062 bp for the BoGM-CSF/VP1 and 1482 bp for the BoIL-2/BoGM-CSF/VP1, respectively. It was further confirmed by PCR with respective primers.

### Characterization of the expressed proteins by SDS-PAGE and Western blot analysis

**Figure 1:**
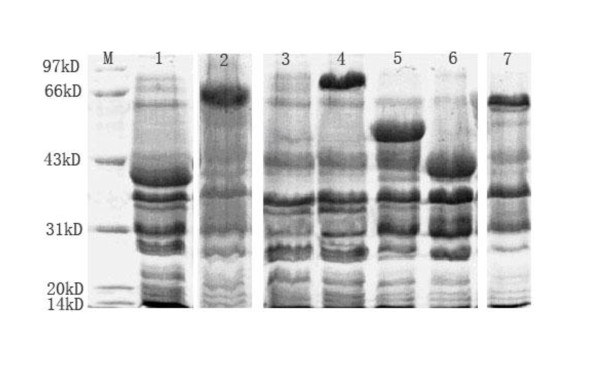
**SDS-PAGE analysis of recombinant protein expressed in BL21**. 20 μl precipitation was sampled from each cultural and analyzed on 15% SDS-PAGE. The results showed that the expressed products were respectively expressed in precipitation with specific target bands of 40 KDa, 66 KDa, 81 KDa, 51 KDa, 41 KDa and 65 KDa, , which were well corresponded to the sizes of BoGM-CSF, BoIL-2/VP1, BoIL-2/GM-CSF/VP, VP1, BoIL-2, BoGM-CSF/VP1. (Lane M: Low molecular weight standard protein marker, Lane 1: BoGM-CSF, Lane 2:BoIL-2/VP1, Lane 3: control, Lane 4: BoIL-2/GM-CSF/VP1, Lane 5: VP1, Lane 6: BoIL-2, Lane 7: BoGM-CSF/VP1).

**Figure 2:**
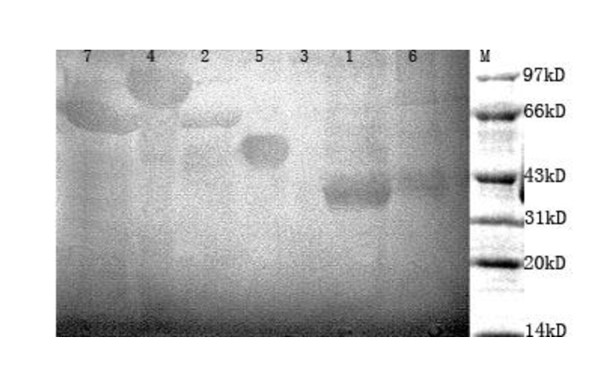
**Western blot analysis of recombinant protein expressed in BL21**. Recombinant proteins were purified and analyzed by Western blot. In Western blot analysis, guinea pig anti- BoIL-2 sera, guinea pig anti-BoGM-CSF sera and bovine FMDV positive sera were respectively used as the primary antibodies, and the expressions of recombinant proteins were all detected with one specific target band, respectively. (Lane M: Low molecular weight standard protein marker, Lane 1: BoGM-CSF, Lane 2:BoIL-2/VP1, Lane 3: control, Lane 4: BoIL-2/GM-CSF/VP1, Lane 5: VP1, Lane 6: BoIL-2, Lane 7: BoGM-CSF/VP1).

To analyze the expressed products, 20 μl samples from the supernatant and precipitation fractions of each culture were analyzed by SDS-PAGE. The result showed that all products were GST fusion proteins and expressed in inclusion body. 40 KDa, 51 KDa, 41 KDa, 65 KDa, 66 KDa, and 81 KDa were observed and represented the sizes of BoGM-CSF, VP1, BoIL-2, BoGM-CSF/VP1, BoIL-2/VP1, BoIL-2/BoGM-CSF/VP1, respectively (Figure 1). The yield of expression for each product is approximately 37% of the total cellular proteins. These constructs were further confirmed by Western-blots (Figure 2).

### Dynamics of serum IgG of FMDV in the inoculated guinea pigs

**Figure 3:**
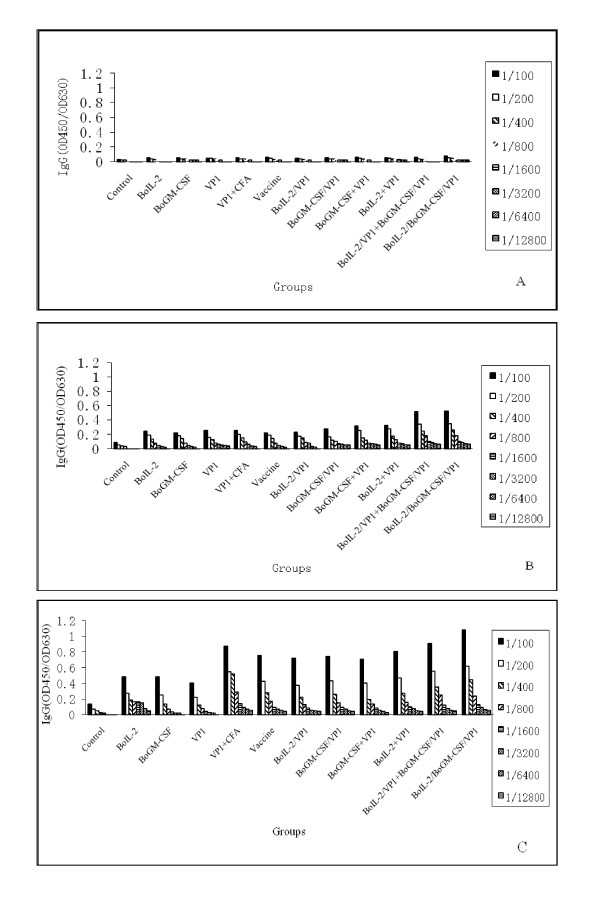
**ELISA analysis of Sera IgG level**. Sera IgG production profile after immunization antibody were analyzed as described in material and methods. The IgG level was determined using ELISA and expressed as OD 450/OD 630. A: Sera IgG level after first immunization, B: Sera IgG level after second immunization, C: Sera IgG level after third immunization.

To evaluate the levels of total IgG against FMDV, the sera obtained from immunized guinea pigs two week after each injection were diluted 1:100 to perform ELISA as shown in Figure 3. The IgG level of serum samples of all groups was increased along with the immunization time. Compared with the control group, sera were detected positive in groups of BoIL-2/BoGM-CSF/VP1, BoIL-2+BoGM-CSF/VP1 and negative in others after the first immunization. After the second and third immunizations, IgG levels were significant higher and increased fast after the third injection in all immunized groups.

Among the groups, IgG levels of BoIL-2/BoGM-CSF/VP1 and BoIL-2/VP1+BoGM-CSF/VP1 groups were statistically significantly higher than those of other groups (P < 0.05). The second high level of IgG was observed in VP1+CFA group, and groups of single cytokine co-expressed or mixed with VP1 and group of vaccine only induced slightly lower level of IgG than VP1+CFA group, but not significantly different. The control groups immunized with BoIL-2 or BoGM-CSF alone induced the lowest level of IgG compared with PBS control group.

### Antigen specific T lymphocyte proliferation assays

**Figure 4:**
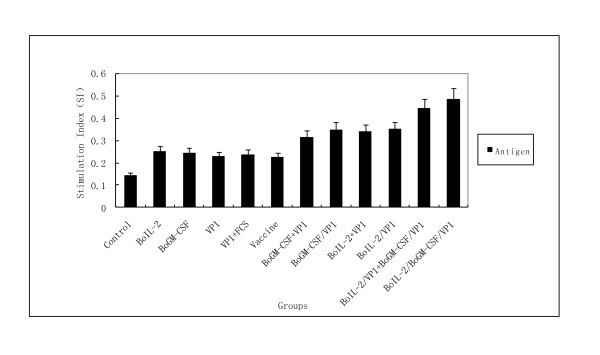
**LAP analysis of T lymphocyte stimulation level**. T lymphocyte proliferation in response to the inoculations with different proteins. T lymphocytes were isolated from the Guinea pig (N = 7) and stimulated with 146 S antigen or unstimulated in vitro, and the stimulation index was defined as the ratio of stimulated wells to unstimulated ones. T cell proliferation responses varied among all the groups.

To determine which cytokine induced better T cell responses, single suspensions of lymphocytes were prepared from guinea pig after the third immunization and assayed with MTT method. As shown in Figure 4 compared with the PBS control group, stimulation indexes (SI) of all groups were increased significantly (P < 0.05). Highest level of proliferation was observed in the group inoculated with BoIL-2/BoGM-CSF/VP1 and followed by the group of BoIL-2/VP1+BoGM-CSF/VP1. The next level of proliferations were observed in four groups given with single cytokine co-expressed or mixed with VP1, followed by VP1 and vaccine groups, but there was no statistically significance with the above four groups.

The result indicated that VP1 plus BoIL-2 and BoGM-CSF could induce significant T cell response, and the combined use of two cytokines had better effect than that of single cytokine as adjuvant. It suggested that these cytokines enhanced the cell-mediated immunity, which was consistent with their known biological function.

### Antigen specific delayed-type hypersensitivity response

**Figure 5:**
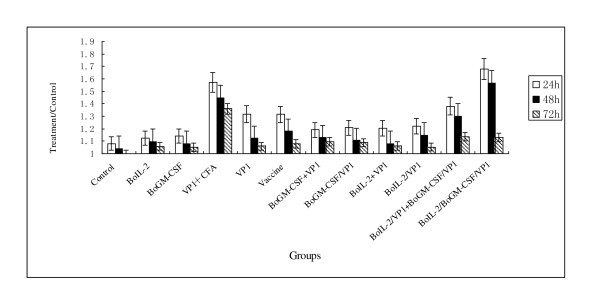
**DTH of Guinea pig inoculated with different proteins**. Fourteen days after the last inoculation, all Guinea pigs (N = 7) were challenged counter-laterally with the 146 S antigen on right footpads as test and saline on left footpads as the negative control. The DTH was defined as the thickness ratio of the right footpad to the left footpad at 24 h, 36 h and 48 h after the challenges.

Delayed-type hypersensitivity (DTH) is a memory immune response and directly reflects the cellular immune response of body. All guinea pigs were treated as described before, and then the thicknesses of footpad were measured respectively at 24 h, 48 h and 72 h. The effects of DTH were assessed by the thickness of left footpad and right footpad ratio. As shown in Figure 5 the highest level of DTH was observed in the group of BoIL-2/BoGM-CSF/VP1, followed by groups of VP1+CFA and BoIL-2/VP1+BoGM-CSF/VP1. The middle level of DTH was seen in the groups of vaccine and VP1, while the DTH level of the four groups that the single cytokine co-expressed or mixed with VP1 were slight lower than the former two groups but no statistically significance. The background level of DTH was from groups of BoIL-2 and BoGM-CSF.

### Th1 and Th2 cytokine profile detected by semi-quantitative RT-PCR

**Figure 6:**
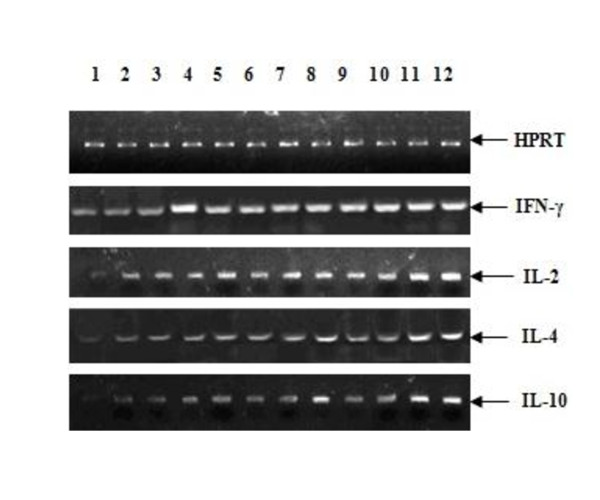
**Semi-quantitative RT-PCR of cytokine gene**. The levels of the Th1 or Th2 cytokines were quantitatively measured by semi-quantitative RT-PCR and showed in Figure 6. For Th1 or Th2 cytokine, mRNA levels were the highest inoculated with the last four groups, followed by co-inoculation with signal cytokine and VP1, VP1 and VP1 + CFA group had the same level with former groups. (1: control, 2: BoIL-2, 3: BoGM-CSF, 4: BoIL-2/VP1, 5: BoGM-CSF/VP1, 6: VP1, 7: vaccine, 8: VP1+CFA, 9: IL-2+VP1, 10: GM-CSF+VP1, 11: BoIL-2/GM-CSF/VP1, 12: BoIL-2/VP1+GM-CSF/VP1).

**Figure 7:**
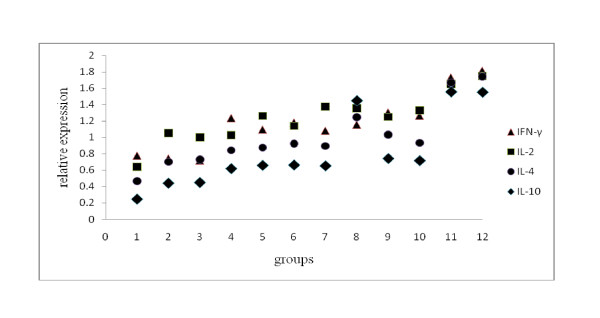
**Cytokine gene relative expression analysis of Semi-quantitative RT-PCR**. Density of electrophoretic bands in Figure 6 were analysed by band leader 3.0. Taking the data of HPRT bands as the background, Th1 and Th2 cytokine relative expression were evaluated by comparing the intensities of their PCR products and showed in Figure 7. For Th1 or Th2 cytokine, mRNA levels were the highest inoculated with the last four groups, followed by co-inoculation with signal cytokine and VP1, VP1 and VP1 + CFA group had the same level with former groups. (1: control, 2: BoIL-2, 3: BoGM-CSF, 4: BoIL-2/VP1, 5: BoGM-CSF/VP1, 6: VP1, 7: vaccine, 8: VP1+CFA, 9: IL-2+VP1, 10: GM-CSF+VP1, 11: BoIL-2/GM-CSF/VP1, 12: BoIL-2/VP1+GM-CSF/VP1).

Cytokines play a dominant role in modulating immune response against infection or in the effectiveness of vaccination. Therefore, semi-quantitative RT-PCR was used to monitor the expression of the representative cytokines. hypoxanthine phosphoribosyl transferase (HPRT), a house-keeping gene, was used as a normalizing control after guinea pigs were immunized. As shown in Figure 6 and Figure 7 the mRNAs of Th1 and Th2 types of cytokines were both evaluated compared with the saline-inoculated group. The groups of BoIL-2/BoGM-CSF/VP1 and BoIL-2/VP1+BoGM-CSF/VP1 showed the highest level of mRNAs of either Th1 or Th2 cytokines. Expression of the cytokines in the groups with single cytokine co-expressed or mixed with VP1 showed the same level of either Th1 or Th2 cytokines as that of groups of Vaccine and VP1. The results indicated that BoIL-2 or BoGM-CSF co-immunized with VP1 could induce both Th1 and Th2 immunity. For the side effects of CFA, group of VP1+CFA showed a higher level of mRNAs of Th2 cytokines than other groups except groups of BoIL-2/BoGM-CSF/VP1 and BoIL-2/VP1+BoGM-CSF/VP1. Groups of BoIL-2 and BoGM-CSF induced the lowest level of cytokines expression.

## Discussion

As an effective cell activator, complete freund's adjuvant could induce humoral immunity and cellular immunity but were restricted to use by serious side effect. In this regard, we examined the effects of cytokine as adjuvant on promoting cellular or humoral immune response. In this study, IL-2 and GM-CSF were selected as adjuvant since they are well-known to induce immune response [12,14] and examined their effects on VP1 subunit vaccination. As a main immunogenic capsid protein of FMDV, VP1 was successfully expressed and co-expressed with two cytokines respectively in *E.coli *BL21 for the subsequent immunizations (Table 1). The result of this study indicated that VP1 alone could induce both humoral and cell-mediated immune response as previously observed [8,9].

**Table 1:** Experiment of groups with different treatment in guinea pigs

Groups	Treatments
Control	PBS
1	BoIL-2
2	BoGM-CSF
3	VP1
4	inactivated FMDV vaccine
5	VP_1_emulsed in the complete fraued's adjuvant (CFA) (VP1+ CFA)
6	Mixture of VP_1_and BoIL-2 (VP1+ BoIL-2)
7	Mixture of VP1 and BoGM-CSF (VP1+ BoGM-CSF)
8	Co-expressed product of BoGM-CSF/VP1
9	Co-expressed product of BoIL-2/VP1
10	Co-expressed product of BoIL-2/BoGM-CSF/VP1
11	Mixture of BoIL-2/VP_1_and BoGM-CSF/VP1 (BoIL-2/VP1+BoGM-CSF/VP1)

In our report, the adjuvant activity of GM-CSF and IL-2 was analyzed. Compared with the VP1 group, groups of GM-CSF/VP1, GM-CSF+VP1, IL-2/VP1 and IL-2+VP1 could induce a much higher IgG level and induce a significant T cell proliferation. It indicated that GM-CSF and IL-2, as adjuvant, could induce both humoral and cell-mediated immune response as for CFA, suggesting that GM-CSF and IL-2 may become the new potent adjuvant, which was consistent with previously documented [18-21]. Interestingly, the results of ELISA and T cell proliferation showed that IL-2 and GM-CSF, combined or mixed with VP1 as adjuvant, induced a similar immune response level, which indicated that IL-2 and GM-CSF co-expressed or co-inoculated with VP1 did not impact their function as adjuvant, which was inconsistent with the results by Shi et al [9].

Cytokines interaction formed regulating network in immune system. In this report, several approaches were used to investigate the combined immune modulating effects of IL-2 and GM-CSF as adjuvant on FMDV vaccination. All results showed that combined use of IL-2 and GM-CSF with VP1 had a better adjuvant effect than single cytokine. It indicated there was synergistic effect between IL-2 and GM-CSF, which was consistent with the previous reports [15,22,23] This may be due to that GM-CSF could attract APC and enhanced the antigen presentation when the VP1 was injected with IL-2 and GM-CSF [24]; IL-2 receptor expression was elevated for the interaction between TCR and antigen [25]. Furthermore, IL-2 could directly enhanced IL-2 receptor expression on antigen selected T cells [26] and could further stimulate the growth and differentiation of those T cells. Interestingly, the adjuvant effect was observed in the BoIL-2/BoGM-CSF/VP1 group rather than in the BoIL-2/VP1+BoGM-CSF/VP1 group, suggesting that IL-2 and GM-CSF co-expressed as adjuvant had a better synergistic effect than co-inoculated with VP1. This probably because, in addition to the suggested synergies, the two fusion cytokines may also had "bridge" function, which could combine surface receptors of T cells, macrophages and DC cell respectively, then formed IL-2/GM-CSF "bridge" in T cells, macrophages and DC cell. This "bridge" could increase the contact of DC and T cell in a short time and the binding of receptor and ligand, therefore, enhancing the antigen-presenting ability of APC, subsequently enhancing the level of cell and humoral immune response, leading to a better adjuvant function than single cytokine. Further experiments are needed to test our hypothetic explanation.

DTH reflected the cell-mediated immune function and especially the manifestation of Th1 type of effect cells. As expected, DTH result was consistent with the results of ELISA and T cell proliferation. It was worth noting that the DTH response level of VP1+CFA group was higher than groups of single cytokine co-expressed or mixed with VP1. The reason for this could be nonspecific stimulation of CFA.

In semi-quantitative RT-PCR, the mRNA levels for IFN-γ, IL-2, IL-4 and IL-10 were measured to assess the profile of cytokines after immunization. Th1 and Th2 type cytokines were all increased after the co-inoculation with recombined proteins in this study, which indicated IL-2 and GM-CSF up-regulated, sequentially, both Th1 and Th2 responses. Groups of BoIL-2/BoGM-CSF/VP1 and BoIL-2/VP1+BoGM-CSF/VP1 could induce the highest expression level of either Th1 or Th2 type cytokines, followed by other groups, which were consistent with the results of ELISA, T lymphocyte proliferation response and DTH. CFA, as the most widely used adjuvant in practical vaccination at present, induced a Th2 subset, which was also reported in other studies [27].

In this report we investigated the ability of IL-2 and GM-CSF as adjuvant to modulate host immune response against FMDV in the controlled experimental conditions. IL-2 or GM-CSF could stimulate cellular and humoral immune response, was a potential adjuvant for the FMDV vaccination. We, for the first time, showed that IL-2 or GM-CSF co-expressed or co-inoculated with VP1 had the equal effect as adjuvant; Two cytokines, GM-CSF and IL-2, when co-expressed with VP1 had a better synergistic effect than that of the co-inoculated. Further evaluation on efficacies and optimizing the immunization pigs and cattle will be the next in our study.

## Conclusions

In summary, the current study indicated the potential for the use of IL-2 and GM-CSF as alternative adjuvant for FMDV vaccination. The study also showed that there was synergistic effect when GM-CSF and IL-2 co-expressed with VP1, which will be useful for further research on FMD vaccines.

## Materials and methods

### Reagents and antigens

RNA isolation and reverse transcription reagent Kits were purchased from Promega (Madison, Wisc., USA), *ExTag *DNA polymerase and all restriction enzymes were purchased from TaKaRa (Dalian, China), BL21 expression vector, pGEX-6p-1, was purchased from Invitrogene, horseradish peroxidase(HRP)-conjugated goat anti-mouse IgG, MTT and TMB were from Sigma(St. Louis, USA). Eight-week-old female guinea pigs were purchased from the Institute of Genetics of Chinese Academy of Sciences.

FMDV O-serotype inactivated vaccine in oil emulsion was acquired from Zhongmu Ltd. (Beijing, China), and the 146 S antigen component was obtained from the purified as described previously and stored at 4°C. The concentration of the 146 s antigen was determined by the Bradford protocol as described previously [28]. 146 S particle contains four major discrete proteins, VP1, VP2, VP3 and VP4. VP1 is the dominant one and provides the major neutralising and T cell epitopes among these four proteins. Therefore, 146 S provides complete antigens/epitopes for the ELISA and T cell proliferation assays.

### Cloning, expression and co-expression of targeted genes

After isolation of peripheral blood mononuclear cells(PBMC) from Holstein cow and stimulated with Con A (10 μg/mg) for 2 h in vitro, total RNA was extracted and reverse transcribed into cDNA by using RNA isolation kit and reverse transcription reagent kit(Promega Inc.) according to the manufacturer's instructions.

The VP1 fragment was amplified from the plasmid PMD18-VP1 (gift from Jin Huali, China Agricultural University). The active mature peptide of BoIL-2 and BoGM-CSF were amplified from cDNA. PCR conditions and primers were indicated as Table 2. The PCR products of BoIL-2, BoGM-CSF and VP1 were purified and digested. All the digested fragments were inserted into the pGEX-6p-1 plasmid respectively, designated as pGEX/BoIL-2, pGEX/GM-CSF and pGEX/VP1.

**Table 2:** Primers for cloning PCR

Target genes*	Primer code	Primers Sequences (5'-3')**	Fragment length	PCR condition
BoIL-2	BoIL-2 F	5' GAA GGA TCC CAC CTC CTA CTT CAA GCT CTA CG 3'	405 bp	94°C for 60 s, 62°C for 60 s and 72°C for 60 s, 35 cycles
	BoIL-2 R	5' CTA GAA TTC CAA GTC ATT GTT GAG TAG ATG C 3'		
BoGM-CSF	BoGM-CSF F	5' CTA GAA TTC GCA CCT ACT CGC CCA CCC AA 3'	378 bp	94°C for 60 s, 62°C for 60 s and 72°C for 60 s, 35 cycles
	BoGM-CSF R	5' TTA CCG CGG CTT CTG GGC TGG TTC CCA G 3'		
VP1	VP1 F	5'GCA CCG CGG ACC ACC TCT GCG GGT GAG TCT 3'	669 bp	94°C for 60 s, 61°C for 60 s and 72°C for 60 s, 35 cycles
	VP1 R	5'GAC CTC GAG CAG AAG CTG TTT TGC GGG T 3'		
	VP1 F1	5'GCA GAA TTC ACC ACC TCT GCG GGT GAG TCT 3'		
	VP1 R1	5'GAC CTC GAG CAG AAG CTG TTT TGC GGG T 3'		

Note: *The GeneBank accession number: BoIL-2 (NM_180997), BoIL-6 (NM_173923), BoGM-CSF (U22385). ** In these primers, sequences for the restriction sites were indicated by underlines.

For the co-expression of BoIL-2 and VP1 in *E. coli*, the VP1 fragment was amplified from the plasmid PMD18-VP1 with the upstream primer VP1 F1 and downstream primer VP1 R1 and digested with EcoRI and XhoI. The IL-2 fragment was subcloned from plasmids pGEX/BoIL-2 and digested with BamHI and EcoRI. The expression vector pGEX-6p-1 was also digested with BamHI and XhoI. All the digested fragments were ligated by T_4 _DNA ligase to yield three constructs, designated as pGEX/BoIL-2/VP1. Between fragments of BoIL-2 and VP1, they were divided by five glycine residues as linker.

For the co-expression of VP1 and BoIL-2, BoGM-CSF in *E. coli*, IL-2, GM-CSF and VP1 were subcloned from plasmids pGEX/BoIL-2, pGEX/GM-CSF and pGEX/VP1. The PCR products of BoIL-2, BoGM-CSF and VP1 were purified and digested respectively. The expression vector pGEX-6p-1 was also digested with BamHI and XhoI. All the digested fragments were ligated by T_4 _DNA ligase respectively, designated as pGEX/IL-2/VP1, pGEX/GM-CSF/VP1, pGEX/BoIL-2/BoGM-CSF/VP1. Between fragments of BoIL-2 and VP1, BoIL-2 and BoGM-CSF, or BoGM-CSF and VP1, they were joined by five glycine residues as linkers.

These constructs were transformed into *E. coli *BL21 in LB plate with 50 μg/ml of Amp+ selection, followed by the identification procedures using both restriction enzyme digestions and PCR. The further confirmation was performed by sequencing analysis.

The confirmed colonies were cultured into LB liquid medium with 50 μg/ml of Amp+ at 37°C until the OD_600 _value reached 0.5. The expression was induced for 6 h with addition of IPTG to achieve a final concentration of 1 mM.

### Characterizations of expressed proteins by SDS-PAGE and Western blot analysis

Sample of 100 μl cultures from each recombinant *E. coli *were homogenized by ultrasonic treatment at 0°C. The protein samples in supernatant and precipitation were subjected in a 15% SDS-PAGE.

The inclusion bodies after ultrasonic treatment were washed three times in 10 mmol/L Tris-Cl buffer (10 mmol/L EDTA, 0.5% Tritonx-100, 0.2 mol/L Urea pH = 8.0)and subsequently washed three times in 10 mmol/L Tris-Cl buffer (10 mmol/L EDTA, 0.5% Tritonx-100, pH = 8.0).Then the inclusion bodies were stepwise dialysed 6 h with Tris-Cl buffer(8 mol/L Urea, 6 mol/L Urea, 4 mol/L Urea and 2 mol/L Urea in each Tris-Cl buffer) and PBS. Purified proteins were collected for Western blot analysis and subsequent immunization.

Purified protein samples were transferred onto the nitrocellulose membrane. The membrane was incubated overnight in 5% bovine serum albumin in Tris-buffered saline-Tween 20 at 4°C before washing for three times in TBS. Subsequently, the membrane was incubated at 37°C for 2 h with the sera of guinea pig anti- BoIL-2, guinea pig anti-BoGM-CSF and bovine FMDV positive sera, diluted 1:1000 in blocking solution. The membrane was washed in TBS and then incubated at 37°C for 2 h with HRP-labeled goat anti-mouse IgG(Sigma), diluted 1:500 in blocking solution. The membrane was washed again and the signals were developed with DAB substrate.

### Immunization and detection of FMDV antibody

Eighty four female guinea pigs were randomly divided into twelve groups (N = 7 per group) as Table 1 and were 2-weeks old at the time of the first immunization. Protein products were injected at the equal total dosage (500 μg per guinea pig, in PBS) by hypodermic multisite injections respectively. Negative control group was injected PBS (100 μl per guinea pig) with the same volume. All test groups were immunized three times with two weeks interval. Sera were collected before vaccination and on the 14^th ^day post each immunization and subsequently analyzed for detection of FMDV antibody.

ELISA plates were used to detect anti-FMDV antibodies in guinea pigs as described previously [8,18]. 146 S antigens (2 μg/ml) were coated on ELISA plates at 4°C overnight and subsequently reacted with sera diluted at 1:100 for 1 h at 37°C. Then sera reacted with 1:1000 diluted goat anti-guinea pigs IgG conjugated with HRP. To detect the ELISA result, colorimetric reaction was developed with TMB (Sigma) and stopped by H_2_SO_4 _and the OD reading was determined at 450 nm/655 nm with a plate reader (Bio-Rad, CA, USA).

### T lymohocyte proliferation

Guinea pigs were immunized as described earlier. Two weeks after final immunization, Guinea pigs were sacrificed and spleens were removed aseptically. Spleen cells were plated at 5 × 10^4 ^cells per well and cultured in triplicate wells for 48 h in presence of 10 μg/ml of 146 s antigen or alone. Culture supernatants were tested to quantify the T cell proliferation as described previously [18]. T lymphocyte proliferation was expressed as stimulation index (SI), which is the ratio of OD_570 nm _of stimulated well (stimulated cell) to OD_570 nm _of unstimulated one [18].

### Antigen specific delayed-type Hypersensitivity (DTH)

Two weeks after the last immunization, Guinea pigs were injected with the 146 S antigen into the right footpads and saline into the left as the negative control. Then the thicknesses of footpads were measured respectively at 24 h, 48 h and 72 h with micrometer to assess the effects of DTH [8,18].

### Semi-quantitative RT-PCR for mRNA of cytokines

Guinea pigs were immunized as described earlier. Two weeks after final immunization, Guinea pigs were sacrificed and spleens were removed aseptically. The lymphocytes were separated from spleens and plated in the 6-well microtiter plate at 5 × 10^4 ^cells per well. The lymphocytes were cultured in triplicate wells with antigen stimulations for 1 h in RPMI-1640 containing 10% FCS. The total RNA was extracted from those cells and the cDNA was synthesized as described above. PCR conditions were optimized with specific primers for the housekeeping gene (HPRT) or cytokine genes indicated as Table 3.

**Table 3:** Primers for Semi-quantitative RT-PCR

Target genes	primers	Fragment length	References
HPRT	5' GTT GGA TAC AGG CCA GAC TTT GTT G 3 GAG GGT AGG CTG GCC TAT GGC T	352 bp	[28]
IL-2	5' TCC ACT TGA AGC TCT ACA G 3' GAG TGA AAT CCA GAA CAT GCC	247 bp	
IFN-γ	5' CAT TGA AAG CCT AGA AAG TCT G 3' CTC ATG GAA ATG CAT CCT TTT TCG	267 bp	
IL-4	5' GAA AGA GAC CTT GAC ACA GCT G 3' GAA CTC TTG CAG GTA ATC CAG G	240 bp	
IL-10	5' CCA GTT TTA CCT GGT AGA AGT GAT G 3' TCT GGT CCT GGA GTC CAG CAG ACT CAA	324 bp	

PCR parameters were performed with minor modifications. Briefly, the PCR mixtures contained 5 μl of PCR buffers, 4 μl of dNTP, 0.5 μl of ExTaq polymerase, 2 μg of cDNAs and 0.5 μl of each primer. The PCR was performed for 32 cycles with parameters of denaturation at 94°C for 1 min, annealing at 60°C for 30 s, extension at 72°C for 1 min, and a final extension at 72°C for 10 min. cDNA from each group was first normalized with the house-keeping gene, HPRT as a reference, each adjusted cDNA was used as template to amplify IFN-γ, IL-2, and IL-4, respectively, according to the conditions described above. All these PCR products were subjected onto electrophoresis on 1.5% of agarose gel and photographed under the UV light [18]. Density of electrophoretic bands in agarose gel were analysed by band leader 3.0. Taking the data of HPRT bands as the background, the relative amount of mRNAs for the cytokine-specific genes was evaluated by comparing the intensities of their PCR products.

### Statistical analysis

Statistical significance between the treatment groups was calculated using One-sided Student's *t-*test and P < 0.05 was considered statistically significant.

## Competing interests

The authors declare that they have no competing interests.

## Authors' contributions

CZ carried out the experiments and wrote the manuscript. BW participated in experimental design and paper revise. MW conceived the studies and participated in experimental design and coordination. All authors read and approved the final manuscript.
